# Umbilical artery thrombosis and maternal positive autoimmune antibodies: two case reports and a literature review

**DOI:** 10.3389/fmed.2023.1187492

**Published:** 2023-06-16

**Authors:** Xin Li, Wei Chen, Tianjiao Liu, Jian Cai, Sumei Wei, Yuhua Du, Chunyan Liu, Zhaolin Gong, Linbo Cheng, Xiaoling Zhou, Min Xiong, Tao Wang, Yalan Li, Xiao Yang, Fan Lai

**Affiliations:** ^1^Chengdu Women's and Children's Central Hospital, School of Medicine, University of Electronic Science and Technology of China, Chengdu, China; ^2^Psychosomatic Medical Center, The Fourth People's Hospital of Chengdu, Chengdu, China

**Keywords:** autoantibody, umbilical artery thrombosis, fetal thrombotic vasculopathy, autoimmune antibodies, pregnant women

## Abstract

**Background:**

Previous studies have shown that abnormal increases in autoimmune antibodies in pregnant women may increase the risk of maternal thrombosis. However, at our hospital, two pregnant women presented with umbilical artery thrombosis and positive maternal autoantibodies were detected in both, which led us to consider whether maternal autoantibodies also played a role in umbilical artery thrombosis.

**Case presentation:**

Case 1: Fetal ultrasound of a 34-year-old pregnant woman at 30^+4^ weeks gestation showed two umbilical arteries, with an inner diameter of approximately 0.15 cm for the smaller was artery. However, only a single umbilical artery blood flow signal was detected. Due to fetal distress, which was noted on abnormal cardiotocography and Doppler ultrasound, an emergency cesarean section was performed at 31^+1^ weeks gestation. The Apgar score of the newborn was 3-8-8. Umbilical cord examination detected thrombosis in the two umbilical arteries. Moreover, blood test results during pregnancy showed nRNP/Sm antibody (+) and SS antibody (+++). Case 2: The first systematic ultrasound of a 33-year-old twin pregnancy at 24^+3^ weeks gestation was normal, but routine fetal ultrasound at 27^+1^ weeks gestation showed only one umbilical artery between fetus A and the placenta. Blood test results showed that the patient was anti-nRNP/Sm antibody (+) in the rheumatoid immune activity test at 27^+3^ weeks gestation. An emergency cesarean section was performed at 34^+6^ weeks gestation because of the single umbilical artery and abnormal maternal coagulation. Both umbilical cords of fetus A and B blood test results showed anti-nRNP/Sm antibody (++). The pathological examination of the umbilical cord and placenta showed the presence of old thrombosis in one of the umbilical arteries of fetus A.

**Conclusions:**

Abnormal maternal autoantibodies may be a risk factor for umbilical artery thrombosis. For these pregnant women, conducting more detailed ultrasound monitoring might get early detection of UAT formation and avoid the occurrence of adverse pregnancy outcomes.

## Background

Umbilical artery thrombosis (UAT) can affect nutrient supply to the fetus, and even endanger the safety of the fetus, resulting in the risk of intrauterine fetal death (1–3). The incidence of UAT is reported to be between 0.025% and 0.045%, but can be up to 0.4% in high risk pregnancies (4).

The cause of UAT is currently unknown. Based on the pathological mechanisms of thrombosis formation, it is believed that blood flow changes, impaired vascular endothelium, and hypercoagulability of the blood are the three common factors that induce thrombosis (5–8). Thus, the hypercoagulable state of maternal blood during pregnancy and umbilical cord compression, caused by various factors, can result in changes in umbilical blood flow and possibly form intra-umbilical thrombosis. There are few reports on the risk factors of umbilical artery thrombosis, and very limited studies include previous adverse pregnancy history, gestational diabetes (GDM), umbilical cord structural abnormalities, and fetal malformation, etc. (9, 10).

Moreover, previous studies have shown that abnormal increases in autoimmune antibodies in pregnant women may increase the risk of maternal thrombosis (11). For example, antiphospholipid antibodies activate the classical complement pathway to induce the production of C5a, interfering with tissue plasminogen activators and plasmin formation; Anti-β2 glycoprotein I antibodies also activate vascular cells, mediate endothelial cell activation, and increase the expression of adhesion molecules, tissue factors, and pro-inflammatory cytokines, thereby increasing the risk of thrombosis (12–16). However, two pregnant women at our hospital who presented with UATs were found to have positive maternal autoantibodies, which led us to consider whether maternal autoantibodies also play a role in UAT.

## Case presentation

### Case 1

**Figure 1:**
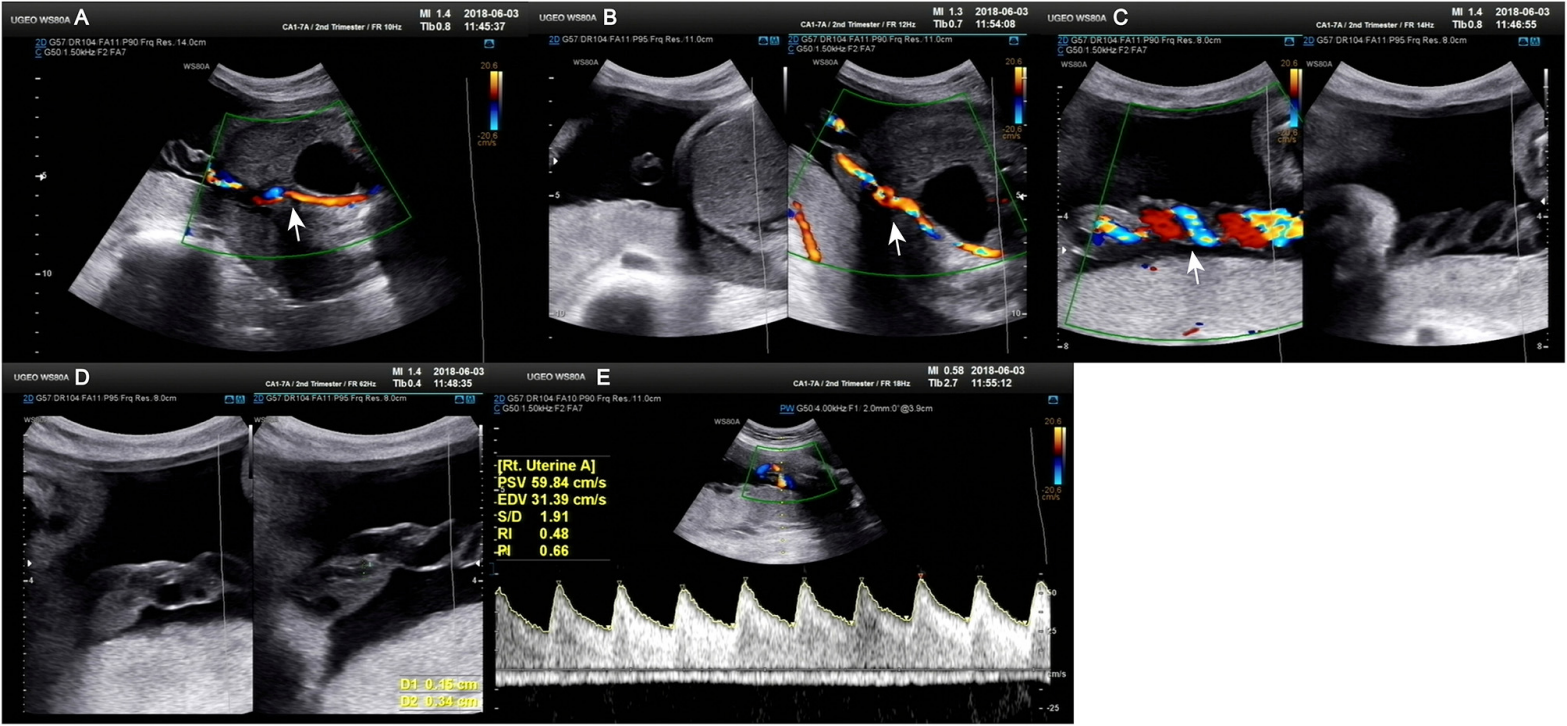
Doppler ultrasound at 30^+4^ weeks gestation showing a single umbilical artery blood flow signal. **(A–C)** Only umbilical vein and single umbilical artery can be seen in ultrasound blood flow signal. **(D)** Structural diagram and vessel width of umbilical artery. **(E)** Various blood flow signal indexes of umbilical artery.

**Figure 2:**
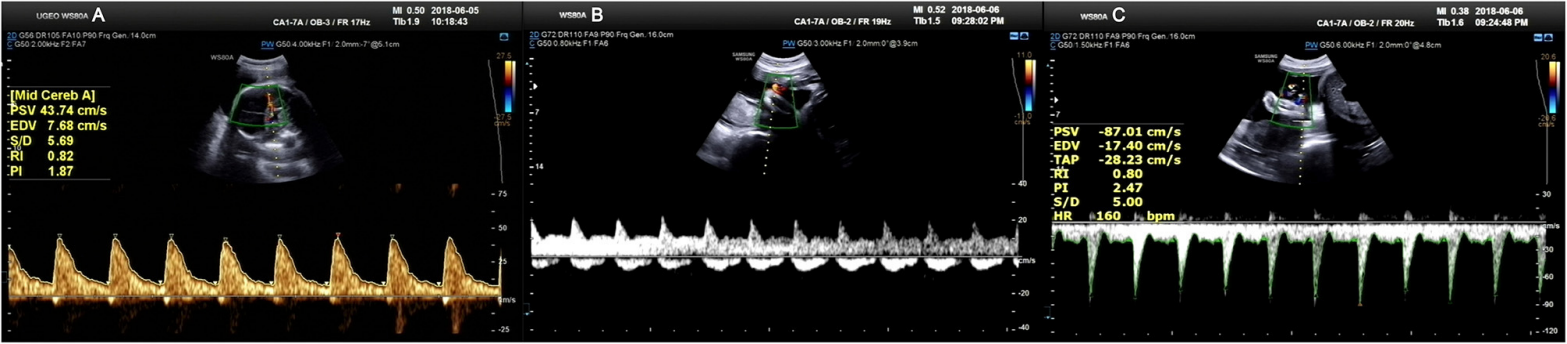
Doppler ultrasound scan at 31^+1^ weeks gestation. **(A)** Various blood flow indexes of the fetal middle cerebral artery. **(B, C)** Various blood flow indexes of fetal umbilical artery.

**Figure 3:**
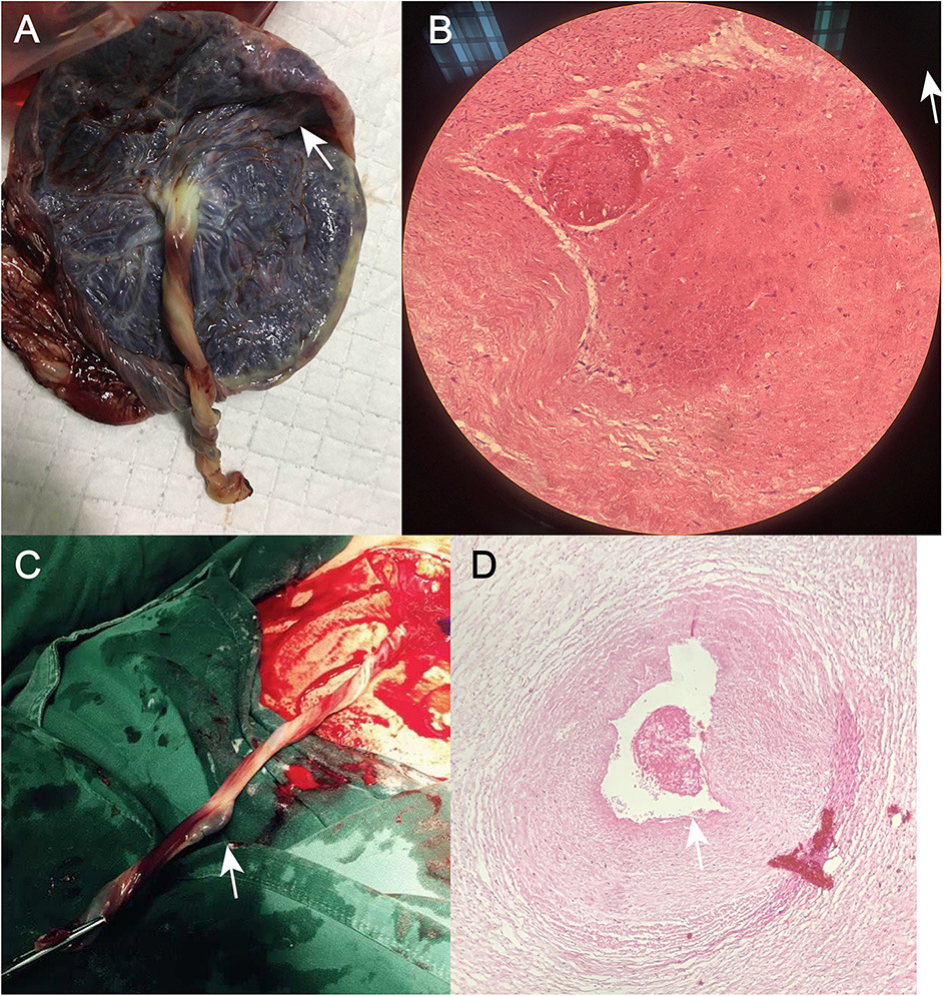
**(A)** Photograph of the umbilical cord after cesarean section in Case 1. **(B)** Postoperative pathological section of umbilical artery thrombosis in Case 1. **(C)** Photograph of the umbilical cord after cesarean section in Case 2. **(D)** Post-operative pathological section of umbilical artery thrombosis in Case 2.

A 34-year-old pregnant woman at 30^+3^ weeks gestation required hospitalization due to decreased fetal movement for 1 day. The patient received no regular prenatal care, amniocentesis, or Down's syndrome screening. The history provided was as follows: gravidity two and parity zero, and a history of stillbirth with unknown cause at 27^+^ weeks gestation by induced vaginal delivery. The patient was diagnosed with gestational diabetes mellitus after admission, and the oral glucose tolerance test result were 5.42-10.67-8.81 (Fasting, 1 h, 2 h) mmol/L. The patient's GDM was controlled through diet, did not use insulin, and the pregnancy blood glucose monitoring results were normal. Routine blood and liver biochemical, kidney and thyroid function tests yielded normal results. The fetus was assessed 2-3 times daily using cardiotocography (CTG) and regularly monitored by Doppler ultrasound (Table 1). Ultrasound at 30^+4^ weeks gestation showed a normal fetal size (umbilical artery systolic/diastolic phase: 2.1, amniotic fluid index: 14.98 cm). Two umbilical arteries seemed to be visualized, and the inner diameter of the thinner artery was approximately 0.15 cm while the other one was 0.34 cm. Nevertheless, only one umbilical artery blood flow signal was detected, where the umbilical cord entered the fetus near the fetal bladder. The fetus was suspected to have a single umbilical artery (Figure 1). Doppler ultrasound demonstrated a pulsating umbilical vein (Figure 2). An emergency cesarean section was performed at 31^+1^ weeks gestation because of fetal distress, which was noted on abnormal CTG and Doppler ultrasound findings. The female newborn weighed 1,815 g with an Apgar score of 3-8-8. Umbilical cord examination showed thrombosis in both umbilical arteries. The umbilical cord, 20 cm in length, showed 12 reverse laps and no entanglement (Figure 3). Blood coagulation and rheumatoid immune activity examinations showed nRNP/Sm antibody (+) and SS antibody (+++). A subcutaneous injection of nadroparin calcium (0.4 mL, 4,000 IU) was administered every 12 h. Blood coagulation parameters were assessed daily over the next 5 days (Table 2). Moreover, the patient underwent tests related to autoimmune diseases, and the results showed that ACL-IgG IgM IgA (-), Sm (-), Ro-52 (-), SS-B (-), Sc1-70 (-), PM-Sc1 (-), Jo-1 (-), CENP B (-), PCNA (-), dsDNA (-), Nukleosomen (-), Nehistone (-), RIB P PRO (-), AMA-M2 (-), nRNP/Sm (+), and SS-A (+++). The patient was diagnosed with antiphospholipid-antibody syndrome. The infant blood coagulation parameters were tested on postpartum days 1 and 3, the results showed a mild increase in INR on the first day of newborn birth and mild decreases in FIB on the first and third day of newborn birth (Table 3).

**Table 1:** Doppler ultrasound and CTG results in Case 1 fetus from 30^+2^ to 31^+1^ weeks of gestation.

**Parameter**	**30^+2^ w**	**30^+3^ w**	**30^+4^ w**	**30^+6^ w**	31^+1^ w^*^
Umbilical artery PI	0.59 (< 5^th^)	0.49 (< 5^th^)	0.74 (< 5^th^)	0.77 (5–50^th^)	2.73 (< 95^th^)
Umbilical artery RI	0.44 (< 5^th^)	0.39 (< 5^th^)	0.52	0.54 (5–50^th^)	0.83
Umbilical artery S/D	1.8 (< 5^th^)	1.63 (< 5^th^)	2.1	2.19 (5–50^th^)	5.91
Middle cerebral artery PI	1.57 (5–95^th^)	1.25 (5–95^th^)	1.81 (50–95^th^)	1.87 (50–95^th^)	0.53 (< 5^th^)
Middle cerebral artery RI	0.77 (5–95^th^)	0.71 (5–95^th^)	0.81 (50–95^th^)	0.82 (50–95^th^)	0.39
Middle cerebral artery S/D	4.41 (5–95^th^)	3.41 (5–95^th^)	5.37 (50–95^th^)	5.69 (50–95^th^)	1.65
Cerebro-placental ratio RI	1.75	1.82	1.56	1.52	0.47
A-wave	Positive	Positive	Positive	Positive	Umbilical veins was pulsating
AFI (cm)	14.98	19	16.43	14.84	14.93
CTG	I	I	I	I	II

AFI, Amniotic fluid index; CTG, cardiotocography (fetal heart rate monitoring); PI, peak systolic velocity; RI, Resistance index; S/D, Systolic/diastolic phase.

CTG I means reactive CTG; CTG II means unreactive CTG.

* Date of cesarean section.

**Table 2:** Maternal blood coagulation parameters in Case 1 before and after administration of anticoagulation therapy.

**Parameter**	**30^+2^ w**	**30^+3^ w**	**30^+4^ w**	**30^+6^ w**	**31^+1^ w**
APTT (s)	25.2↓	30.3	28.9	36.2	38.3
PT (s)	10.5	11.7	10.80	12.7	12.9
INR	0.88	0.85	0.91	0.94	0.96
FIB (g/L)	5.23↑	5.28↑	5.42↑	6.26↑	5.29↑
TT (s)	12.1	16.6	11.70	17.4	19.2
D-dimer (ug/ml)	N/A	8.06↑	N/A	2.08↑	2.00↑
FDP (ug/ml)	N/A	27.01↑	N/A	6.64↑	8.12↑

^*^Day of the cesarean section.

APTT, activated partial thromboplastin time; D-D, D-dimer; FIB, Fibrinogen; INR, international normalized ratio; N/A, not assessed; PT, determination of plasma prothrombin time; TT, prothrombin time; FDP, fibrin degradation products. Arrows indicate elevation above the normal range.

**Table 3:** Infant blood coagulation parameters in Case 1.

**Parameter**	**First day of birth**	**Third day of birth**
APTT (s)	62	50.3
PT (s)	22	16.4
INR,	1.86↑	1.38
FIB (g/L)	1.09↓	1.98↓
TT (s)	21.20	11.20

APTT, activated partial thromboplastin time; FIB, Fibrinogen; INR, international normalized ratio; PT, determination of plasma prothrombin time; TT, prothrombin time. Arrows indicate values below the normal range.

### Case 2

A 33-year-old woman with twin pregnancy at 34+4 weeks gestation required hospitalization after detecting a single umbilical artery and umbilical cord entanglement in fetus A, and abnormal maternal coagulation. Upon admission, the following were noted: 1. Dichorionic twin pregnancy; 2. Chronic hepatitis B surface antigen virus carrier; 3. *In vitro* fertilization embryo transfer pregnancy. The patient underwent regular obstetric examinations throughout the pregnancy, and no obvious abnormalities were found in maternal thyroid function and glucose tolerance tests, nuchal translucency, non-invasive DNA testing, systematic ultrasound, and cardiac ultrasound (Table 4). The initial systematic ultrasound of the fetus at 24^+3^ weeks gestation showed no abnormalities, but routine fetal ultrasound at 27^+1^ weeks gestation showed only one umbilical artery between fetus A and the placenta. The patient's blood test results showed anti-nRNP/Sm antibody (+), and protein S and antithrombin III levels were decreased at 27^+3^ weeks gestation, and was prescribed 0.4 mL daily enoxaparin sodium subcutaneous injection and 75 mg oral aspirin. At 32^+3^ weeks gestation, anti-nRNP/Sm antibodies (++) were rechecked in the rheumatoid immune activity test (Table 5). At 34^+4^ weeks gestation, the fetal routine ultrasound suggested a single umbilical artery, with the umbilical cord wrapped around the fetus's neck, and a “U”-shaped induration noted on the fetal neck. It was recommended that a cesarean section should be performed at 34^+6^ weeks gestation. Fetus A was found to be a boy with a weight of 2,510 g and an Apgar score of 10-10-10. Both fetus A and B umbilical cord blood test results showed anti-nRNP/Sm antibody (++). Pathological examination of the umbilical cord and placenta showed the presence of old thrombosis in one of the umbilical arteries of fetus A, multiple pseudoknots of the distal umbilical cord, and surrounding interstitial edema (Figures 3C, D). Placentae A and B were found to have small amounts of inflammatory cell infiltration of the fetal focal areas, uneven maturity of the placental villi, and villous edema in some areas, of which placenta B also showed violar space thrombosis, with the focal area seen in individual vascular villus.

**Table 4:** Doppler ultrasound and CTG results in Case 2 fetus A from 27^+1^ to 34^+6^ weeks of gestation.

**Parameter**	**27^+1^ w**	**29^+1^ w**	**31^+1^ w**	**33^+1^ w**	**34^+4^ w**	**34^+5^ w**	**34^+6^ w**
Umbilical artery PI	0.79 (5–50^th^)	0.65 (< 5^th^)	0.69 (< 5^th^)	0.7 (?5^th^)	0.77 (5–50^th^)	0.78 (5–50^th^)	0.79 (5–50^th^)
Umbilical artery RI	0.53	0.5	0.52	0.49	0.54	0.54	0.53
Umbilical artery S/D	2.13	2	2.08	1.97	2.19	2.18	2.12
Middle cerebral artery PI	1.84 (5–50^th^)	1.68 (5–50^th^)	1.86 (50–95^th^)	1.93 (50–95^th^)	1.62 (5–50^th^)	1.65 (5–50^th^)	1.63 (5–50^th^)
Middle cerebral artery RI	0.84	0.8	0.83	0.85	0.78	0.8	0.8
Middle cerebral artery S/D	6.1	4.89	6	6.64	4.58	4.9	4.95
Cerebro-Umbilical ratio PI	2.33	2.58	2.69	2.75	2.1	2.11	2.06
AFV, cm	5.8	3.9	6.8	4.9	5.1	4.7	4.1

AFV, Amniotic fluid vertical depth; PI, peak systolic velocity; RI, Resistance index; S/D, Systolic/diastolic phase.

**Table 5:** Maternal blood coagulation parameters in Case 2 after anticoagulation therapy.

**Parameter**	27^+3^ w^*^	**32^+3^ w**
APTT (s)	30.3	24
PT (s)	11.4	11
INR	0.99	0.95
FIB (g/L)	4.4↑	4.97↑
TT (s)	18.3	16
D-dimer (ug/ml)	2.34↑	2.21↑
FDP (ug/ml)	4.75	4.78
PC (%)	90.7	N/A
PS (%)	29.4↓	N/A
ATIII	66.2↓	N/A

APTT, activated partial thromboplastin time; FIB, Fibrinogen; INR, international normalized ratio; PT, determination of plasma prothrombin time; TT, prothrombin time; FDP, fibrin degradation products; PC, Protein CPS; Protein S; ATIII, Antithrombin III. Arrows indicate values below the normal range.

* Before using anticoagulation therapy.

## Discussion

UAT can be diagnosed clinically, histologically, or by ultrasound. However, before delivery, only ultrasonography can be performed, which is likely to lead to misdiagnosis. Klaritsch et al. reported a case of misdiagnosis where a UAT was mistaken for a single umbilical artery (7). Umbilical artery automatic atresia will also present with a single umbilical artery, with normal fetal development, which is difficult to distinguish from UAT. Single umbilical arteries are the main cause of UAT misdiagnosis.

Ultrasonography in Case 1 showed what appeared to be two umbilical arteries, but only a single blood flow signal was detected, leading to the diagnosis of a single umbilical artery. Case 2 showed a change from the initial two umbilical arteries on color Doppler ultrasound to a single umbilical artery, suggesting the possibility of an arterial embolism. Reports have proposed the classis presentation of the “orange grabbed sign” (17), which helps in is easy distinguishment between congenital single umbilical arteries and UAT, and can reduce the incidence of UAT misdiagnosis in ultrasonography (11, 17).

Fetal UAT is associated with a variety of adverse clinical outcomes, such as spontaneous intrauterine death, stillbirth, fetal growth restriction, neonatal asphyxia, and fetal intracranial hemorrhage. The cause of UAT is unknown, and both patients showed abnormalities in antinuclear antibodies (ANAs) with UAT. This possibly suggests that abnormal maternal ANAs may be associated with the incidence of UAT. Studies have shown that ANAs are related to the body's inflammatory responses and atherosclerosis (18). ANAs may be involved in vascular wall integrity changes together with vascular endothelial-related factors. It can thus be speculated that abnormal maternal ANAs may cause damage to the umbilical artery wall and induce thrombosis. At the same time, with typical autoimmune diseases, such as systemic lupus erythematosus, vasculitis is a common pathological change. Severe vascular lesions can lead to vascular lumen stenosis or even occlusion, causing ischemia and necrosis of vascular supply tissue. These pathological changes in the vessels are closely related to the inflammatory response and coagulation and fibrinolytic system imbalance that is induced by autoimmune antibodies (19). Moreover, the presence of the “lupus anticoagulant” in maternal blood is associated with thrombosis in the placental vessels and a high rate of fetal loss (20). This suggests a potential correlation between autoimmune antibody abnormalities and UAT risk. In addition, the Case 1 women have GDM during pregnancy. Previous studies showed that GDM might be also a risk factor for UAT (9).

In terms of treatment, considering the pathological changes that ANAs may induce, and the single umbilical artery noted on color ultrasound, according to previous study and consensus among some Chinese experts, early anticoagulant treatment with LWMHs may improve pregnancy outcomes (21). However, there may be a risk of sudden fetal death in the uterus during expectant treatment. So, it is necessary to have sufficient communication with the patient and closely monitor the fetal condition. If there is serious progress in the condition, immediately terminate the pregnancy through cesarean section.

This preliminary study has strengthened our understanding of the potential effect of maternal positive autoimmune antibodies on UAT, but also has several limitations that need to be acknowledged. Firstly, Case 1 female has GDM, which may be also a risk factor for UAT and might have an impact on the occurrence of UAT in Case 1. Secondly, since this study is retrospective, Case 2 occurred in 2019, and only the patient's serological test data can be seen in our medical record system. Therefore, there are omissions in the color Doppler ultrasound data of this patient. Thirdly, based on current evidence, we are indeed currently unable to confirm the inevitable correlation between umbilical artery thrombosis and maternal positive autoimmune antibodies. However, there is currently too little study on the risk factors of UAT, and due to the rarity and high risk of UAT, reporting on any potential influencing factors should be meaningful. Both of our patients showed positive autoimmune antibodies, and it is indeed possible that positive autoimmune antibodies may cause the formation of UAT in pathological mechanisms. Therefore, we believe that our reported results are meaningful and can provide potential references for further elucidating the mechanism of UAT occurrence. However, due to the small sample size from a single medical center, the preliminary results of this study should be validated in a prospective multicenter study with a larger sample size.

## Conclusions

Considering these two case reports and the literature review, abnormal maternal autoantibodies may be a risk factor for UAT. For these patients, conducting more detailed ultrasound monitoring might get early detection of UAT formation and avoid the occurrence of adverse pregnancy outcomes.

## Data availability statement

The original contributions presented in the study are included in the article/Supplementary material, further inquiries can be directed to the corresponding authors.

## Ethics statement

The study was approved by the Ethics Committee of the Chengdu Women's and Children's Central Hospital (No. 201830). The patients provided consent for all the procedures. Written informed consent has been obtained from the individuals for the publication of any potentially identifiable images or data included in this article. Written informed consent was obtained from the patients for the publication of this case report.

## Author contributions

XY designed the research protocol. FL and XL conducted the study and drafted the manuscript. FL analyzed the data. TL, JC, SW, YD, CL, ZG, LC, XZ, MX, TW, and YL critically revised the manuscript. XY and FL provided funding resource. All authors have accepted responsibility for the content of the final manuscript and approved submission.
