# Supplementary material for: Umbilical artery thrombosis and maternal positive autoimmune antibodies: two case reports and a literature review
Source: Front Med (Lausanne). 2023 Jun 16;10:1187492. doi: 10.3389/fmed.2023.1187492 (PMC10313525; doi:10.3389/fmed.2023.1187492)
Supplement: Supplementary file 1 [file Data_Sheet_1.docx]

**Supplementary table 1** Risk factors and prognosis data of UAT in previous studies

| Author | Risk factors | Prenatal ultrasound signs | Monitoring | Treantment | Adverse pregnancy outcomes* |
| --- | --- | --- | --- | --- | --- |
| Xiafang Wu | Abnormal pregnancy history, GDM, umbilical cord abnormality | UAT | ultrasound and CTG | Expecting treatment/ Emergency operation | 50% |
| P. Klaritsch | Umbilical cord abnormality | UAT | - | Emergency operation | 0% |
| Y Sato | Fetal malformation | UAT | ultrasound and CTG | Expecting treatment/ Emergency operation | 81.80% |
| Jing Wei | Fetal malformation, Umbilical cord abnormality | UAT | ultrasound and CTG | Expecting treatment/ Emergency operation | 37.50% |
| Caixia Han | Umbilical cord abnormality | UAT | ultrasound and CTG | Expecting treatment | 0% |
| Christine Shilling | Fetal malformation, Umbilical cord abnormality | UAT | - | - | 100.00% |
| Kei Tanaka | Umbilical cord abnormality | Orange grabbed sign | ultrasound | Expecting treatment | 0% |
| Gustavo Henrique de Oliveira | Umbilical cord abnormality | UAT | ultrasound and CTG | Expecting treatment | 100.00% |
| Ting Wang | Fetal malformation, Umbilical cord abnormality | UAT | ultrasound and CTG | Expecting treatment/ Emergency operation | 50% |

*Intrauterine fetal death, severe neonatal asphyxia, neonatal malformations, long-term complications, or neonatal death

CTG: cardiotocography( fetal heart rate monitoring), UAT: umbilical artery thrombosis
